# *APOL1* renal risk variants promote cholesterol accumulation in tissues and cultured macrophages from APOL1 transgenic mice

**DOI:** 10.1371/journal.pone.0211559

**Published:** 2019-04-18

**Authors:** Jung-Hwa Ryu, Mengyuan Ge, Sandra Merscher, Avi Z. Rosenberg, Marco Desante, Hila Roshanravan, Koji Okamoto, Myung K. Shin, Maarten Hoek, Alessia Fornoni, Jeffrey B. Kopp

**Affiliations:** 1 Kidney Disease Section, NIDDK, NIH, Bethesda, Maryland, United States of America; 2 Katz Family Division of Nephrology and Hypertension and Peggy and Harold Katz Drug Discovery Center, University of Miami School of Medicine, Miami, Florida, United States of America; 3 Dvision of Pathology, Johns Hopkins School of Medicine, Baltimore, Maryland, United States of America; 4 Merck & Company, Inc., Kennilworth, New Jersey, United States of America; UCL Institute of Child Health, UNITED KINGDOM

## Abstract

Apolipoprotein L1 (*APOL1*) genetic variants G1 and G2, compared to the common allele G0, are major risk factors for non-diabetic kidney disease in African descent populations. APOL1 is a minor protein component of HDL, as well as being expressed in podocytes and vascular cells. Reverse cholesterol transport involves the transport of cholesterol to HDL by cellular ATP-binding cassette; ABCA1 and ABCG1 with subsequent delivery from peripheral tissues to the liver. With impaired reverse cholesterol transport, lipid accumulation occurs and macrophages morphologically transform into foam cells, releasing inflammatory factors. We asked whether the APOL1 risk variants alter peripheral cholesterol metabolism and specifically affect macrophage cholesterol efflux. Tissues and bone marrow (BM)-derived monocytes were isolated from wild-type mice (WT) and from BAC/APOL1 transgenic (APOL1-G0, APOL1-G1, and APOL1-G2) mice, which carry a bacterial artificial chromosome that contains the human APOL1 genomic region. Monocytes were differentiated into macrophages using M-CSF, and then polarized into M1 and M2 macrophages. Cholesterol content, cholesterol efflux, and ABCA1 and ABCG1 mRNA expression were measured. Kidney, spleen, and bone marrow-derived macrophages from APOL1-G1 and -G2 mice showed increased cholesterol accumulation and decreased ABCA1 and ABCG1 mRNA levels. BM-derived macrophages from APOL1-G1 and -G2 mice showed significantly reduced cholesterol efflux compared to WT or APOL1-G0 macrophages. Taken together, the evidence suggests that APOL1-G1 and -G2 risk variants impaired reverse cholesterol transport through decreased expression of cholesterol efflux transporters suggesting a possible mechanism to promote macrophage foam cell formation, driving inflammation in the glomerulus and renal interstitium.

## Introduction

Genetic variants in *APOL1*, encoding apolipoprotein L1, constitute a major cause of glomerular disease in individuals with sub-Saharan African ancestry. These diseases include focal segmental glomerulosclerosis [1], collapsing glomerulopathies in various settings including HIV-associated nephropathy [1], and arterionephrosclerosis. *APOL1* variants, termed G1 and G2, are associated with faster progression to end-stage kidney disease in these disorders, as well as in lupus nephritis. The *APOL1* G1 renal risk variant is comprised of two coding variants (rs73885319, p.S342G) and rs6091914 (p.I384M). The *APOL1* G2 renal risk is an deletion variant (rs71785313, p.NYK388K). More recently efforts have suggested a role of the *APOL1* kidney disease variants in cardiovascular disease as well [2]

Reverse cholesterol transport contributes to the regulation of cholesterol levels in peripheral tissues, including tissue macrophages, by returning cholesterol to the liver, Reverse cholesterol transport involves efflux of free cholesterol from macrophages or other cells to apolipoprotein A-1 (ApoA-1) and HDL through membrane ATP-binding cassette transporter A1 (ABCA1) and G1 (ABCG1). Impaired cholesterol efflux from macrophages is a better predictor of cardiovascular disease than are levels of HDL cholesterol, and is independent of HDL cholesterol [3]. Recently, a role for cholesterol accumulation in glomerular injury has been demonstrated, using mouse models of Aport syndrome and focal segmental glomeruluosclerosis treated with a cholesterol-depleting agent [4].

We have explored the possibility that *APOL1* genetic variants influence transcellular cholesterol trafficking, using kidneys, spleens, and macrophages derived from monocytes from BAC/APOL1 transgenic mice. Only certain primates possess the *APOL1* gene and therefore mice lack the *APOL1* gene.

## Materials and methods

### BAC/APOL1 transgenic mice

Mice were generated by microinjecting a ~47.2 kb fragment, encompassing the human *APOL1* gene, part of the *APOL2* gene (exons 1 and 2) and part of the *MYH9* gene (exons 39–41), from human RPCIB-753 BAC library (sequence location: chromosome 22:36237712–3628488), as illustrated in S1 Fig. RT-PCR analysis demonstrated that the transgene was expressed in kidney (data not shown), spleen and macrophages isolated from spleen (S2 Fig).

To induce renal injury, mice were subjected to a combination of four interventions: uninephrectomy, normal saline as the only oral fluid for two weeks, combined with slow release deoxycorticosterone acetate pellet and slow release aldosterone pellet (both rom Innovative Research of America, Sarasota, FL) during that period. These interventions induce systemic hypertesion and hypertensive renal injury, including glomerulosclerosis.

Histopathologic examination and analysis of the blood and urine chemistry indicate that all three lines of mice lack pathologic findings compared to age-matched wild-type mice of the same strain. Immunoflouresence staining was performed on frozen sections for APOL1 (polyclonal rabbit antibody, Sigma, St Louis, MO, HPA01885) and podocytes stained for podocalyxin (monoclonal antibody, R&D Biosystems, Minneapolis, MN, AF1556). APOL1 protein expression levels were generally similar in mouse serum (S3 Fig), kidney glomeruli (S4 Fig and liver (S5 Fig) across the variants with the APOL1-G2 protein levels were reduced in liver (anti-APOL1 antibody By contrast, there was minimal *APOL1* transgene expression in lung (S6 Fig).

Wild type (WT) mice on a 129 background and BAC/APOL1-G0, -G1 and -G2 transgenic mice were bred and kept in specific pathogen-free conditions at National Institutes of Health (NIH). All mice in study were male, aged 8–9 week. Thirty-two (32) male mice were studied in the experiments descscribed here. Animal experimental procedures were approved in advance by the NIDDK Animal Care and Use Committee. Animal care adhered to the NIH guidelines for the Care and Use of Laboratory Animals; 32 mice were used in total. Mice were maintained in temperature-controlled condions, with free access to water and food. Interventions were performed under anesthesia by Avertin (2, 2, 2-tribromoethanol, Sigma-Aldrich). After anesthesia, mouse blood was taken cardiac puncture using 27-gauge needle syringe. Kidneys, spleen, and both lower extremities were harvested.

#### Differentiation of M1 and M2 macrophage from BAC/APOL1 bone marrow-derived monocytes

Bone marrow cells were harvested from femurs and tibias of mice. Macrophage differentiation was performed as previously described [5]. Bone marrow-derived monocytes were isolated using commercial monocyte isolation kit (Miltenyi Biotec, San Diego, CA) and counted and plated at a density of 2X10^5^ cells/well of 24-well plate. Cells were cultured in complete RPMI 1640 (Thermo Fisher Scientific, Waltham, MA); 10% heat-inactivated fetal bovine serum (FBS) (Corning, Corning, NY), 1% penicillin/streptomycin, 1% glutamine containing 30 ng/mL macrophage colony-stimulating factor (M-CSF, PeproTech, Rocky Hill, NJ) in 5% CO_2_ at 37°C. The medium was replaced on days 3 and 6. On day 7, the cells were washed and stimulated with IFN-γ (20 ng/mL, PeproTech, Rocky Hill, NJ) for induction of M1 macrophages, alternatively with IL-4 (20 ng/mL, PeproTech) for induction of M2 macrophages for 48 hours. The cells were harvested on day 9 for experiments described below.

### [^3^H]-Cholesterol efflux assay in murine BAC/APOL1 macrophages

We performed cholesterol efflux assay using [^3^H]-cholesterol differentiated M1 and M2 macrophages, which were washed with sterile PBS three times and then labeled with 0.5 μCi/mL of [^3^H]-cholesterol (PerkinElmer, Waltham, MA, USA) in phenol-free RPMI medium and incubated at 37°C for 18 hours. The cells were washed with PBS and equilibrated for 4 hours at 37°C in RPMI medium containing 2% serum from wild type mice as a cholesterol acceptor. The medium was collected and filtered using vacuum filtration system. The cells were lysed in 0.5% SDS for 2 hours at room temperature. Each cell lysate and medium was mixed with scintillation solution and radioactivity of [^3^H]-cholesterol in each cell lysate and medium was determined by scintillation beta counter (PerkinElmer, Waltham, MA, USA). The cholesterol efflux rate was calculated by disintegration per minute of [^3^H]-cholesterol in medium/([^3^H]-cholesterol in medium + [^3^H]-cholesterol in cell lysate) x 100.

### Establishment of APOL1 G0, APOL1 G1 and APOL1 G2 overexpressing HeLa cells

HeLa cells were stably infected with lentivirus carrying the *APOL1* risk variants G0, G1 and G2 under the CMV promoter. Cells were maintained and expanded in at 37^0^ C in DMEM with 10% FBS in the presence of 5% CO_2_.

### Determination of cellular cholesterol content using Bodipy BODIPY493/503 staining

HeLa cells transfected with different *APOL1* alleles were cultured, fixed with PFA and stained with Bodipy493/503 to determine changes in the neutral lipid droplet content due to ApoL1 variant expression and cell-mask blue (Invitrogen) to identify individual cells. The Opera High Content Screening system and Acapella Image Analysis software were used to determine the number of Bodipy 493/503 positive lipid droplets per cell.

### [^3^H]-Cholesterol efflux assay in HeLa cells

To measure cholesterol efflux from HeLa cells, a previously described method was used with slight modification[6]. Briefly, cells were labeled with 1 nCi/ml [^3^H]-cholesterol in medium with 1% FBS for 24 hours, washed in PBS, incubated in DMEM containing 20 μg/ml delipidated APOA1 or 2% human serum for 6 hours. HeLa cells were purchased from the American Type Collection, Manassas, VA. Pooled normal human serum used for cholesterol efflux assays in HeLa cells was purchased from Innovative Research, Novi, MI (Cat. No. IPLA-SER).

Cholesterol efflux to medium was determnined by measurement of radioactivity in an aliquot of medium by scintillation counting. Cholesterol efflux to medium was expressed as percent of total cholesterol.

### Cholesterol quantitation

Total cholesterol amount in kidney, spleen, and bone marrow-derived macrophages from BAC/APOL1 mice were measured using a cholesterol quantitation kit (Sigma-Aldrich, St. Louis, MO) using colorimetric method according to the manufacturer’s instructions.

### Oil Red O stain

Sub-confluent macrophages plated in 24-well tissue culture plates were incubated with 50 μg/mL of aggregated LDL for 24 hours in RPMI medium, after that, the medium was completely removed and cells were rinsed with PBS three times. Subsequently, cells were fixed in 10% phosphate buffered formalin (Sigma-Aldrich) for 1 hour and washed with 60% isopropanol. Completely dried cells and tissue frozen sections (following brief formalin fixation) were stained with Oil Red O (filtered 0.5% Oil Red O in 60% isopropanol) for 1 hour. The cells were extensively washed with dH_2_O until the grossly-appeared red color in each well was absolutely disappeared and examined by light microscopy.

### RNA isolation and reverse transcriptase PCR

Total RNA was manually extracted from tissues of kidney, spleen and macrophages using RiboZol RNA extraction reagent (AMRESCO, Cleveland, OH) and subsequent total RNA isolation was conducted using RNA isolation kit (Macherey-Nagel, Bethlehem, PA). Purified RNA concentration was measured by spectrophotometry using Nanodrop (Thermo Fisher Scientific, Wilmington, DE). One microgram of RNA was reverse transcribed using GoScrip Reverse Transcription PCR System (Promega, Madison, WI) according to the manufacturer’s protocol. For HeLa cells, RNA was extracted using the RNeasy Mini Kit (Qiagen, Hilden, Germany). Reverse transcription was performed using the qScript cDNA SuperMix (Quanta, Gaithersburg, MD) according to the manufacturer’s protocols.

### Real-time quantitative PCR

For real-time quantitative PCR for ABCA1, ABCG1 mRNAs from kidney, spleen, macrophages, the primers for each gene were purchased from Life Technologies, Inc. (Gaithersburg, MD): ABCA1 sense, 5’-TGACATGGTACATCGAAGCC-3’; ABCA1 antisense, 5’-GATTTCTGACACTCCCTTCTGG-3’; ABCG1 sense, 5’-TCAAGGACAATGCGTATACAGG-3’; ABCG1 antisense, 5’-TGTTCTGATCCCCGTACTCC-3’; GAPDH sense, 5’-CCGCATCTTCTTGTGCAG-3’; and GAPDH antisense, 5’-TGCCGTGAGTGGAGTCATAC-3’. Quantitative PCR reactions were performed in RT-PCR thermal cycler (QuantStudio, Thermo Fisher Scientific, Waltham, MA) using FastStart SYBR Green Master mix (Roche, Indianapolis, IN) according to manufacturer’s instructions. RT-PCR using cDNA isolated from HeLa cells was performed using the StepOnePlus system (Thermo Fisher Scientific, Wilmington, DE) with PerfeCTA SYBR Green FastMix (Quanta, Gaithersburg, MD). The primer sequences for APOL1 were: APOL1 sense, 5’-CCGGGTCACTGAGCCAATC-3’; APOL1 antisense, 5’-ACACGAGGTAGACTACATCCAG-3’. The relative expressed levels of each mRNA were normalized to mRNA levels of GAPDH.

**Fig 1:**
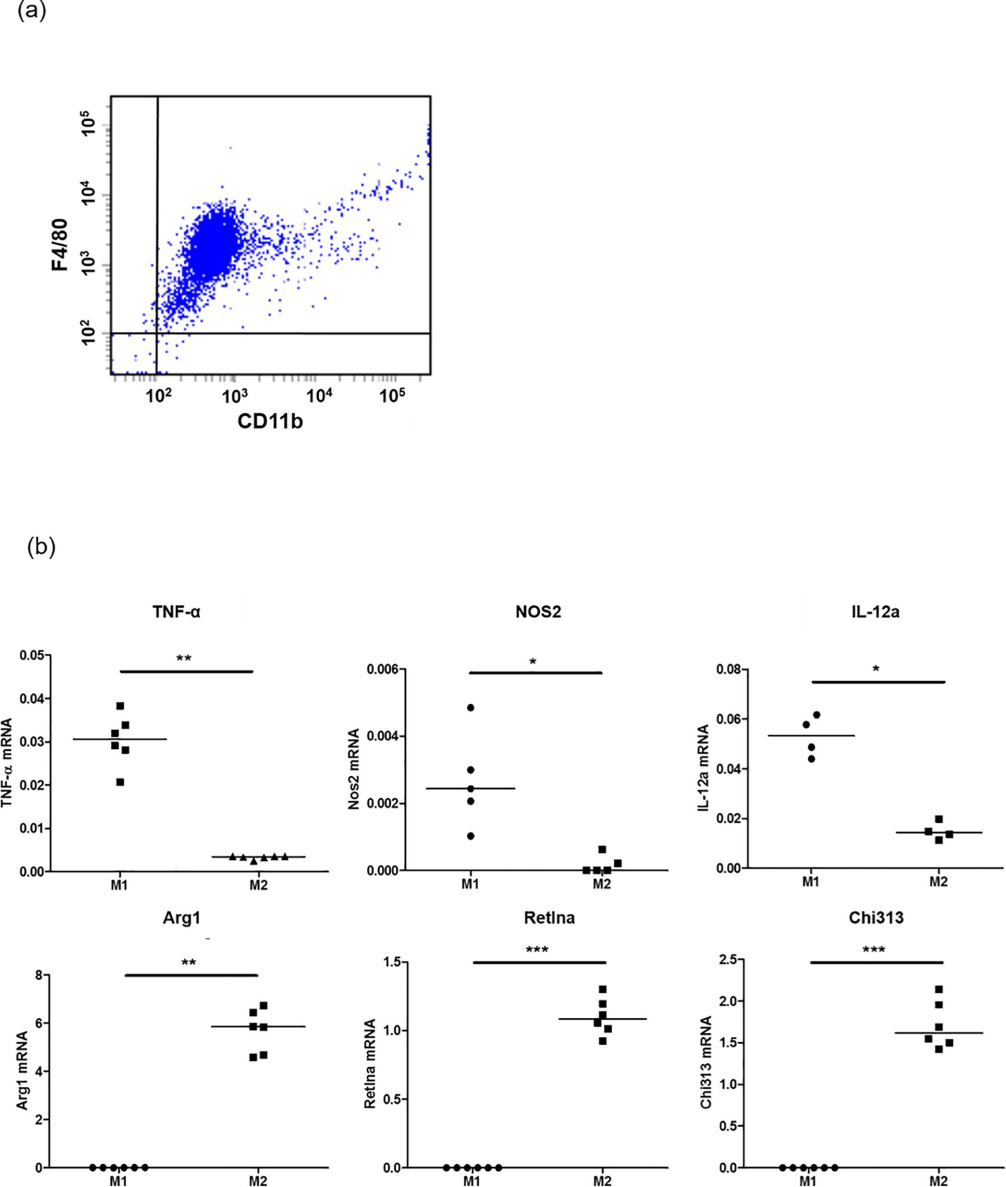
Identification of M1 and M2 macrophages. Panel (a). Murine bone marrow-derived macrophages were analyzed by flow cytometry using antibodies against F4/80 and CD11b. F4/80^+^/CD11b^+^ cells are analyzed as macrophages. Panel (b). Using quantitative RT-PCR, M1 macrophages were identified by expression of *Tnf*, *Nos2*, and *IL-12a*, t and M2 macrophages were identified by expression of *Arg1*, *Retnla*, and *Chi313* Mann-Whitney U-test; *P < 0.05, **P < 0.001, ***P < 0.0001.

To evaluate the adequate polarization of macrophages into M1 and M2 subtypes, the F4/80^+^CD11b^+^ cells obtained by flow cytometry were analyzed for mRNA expression of characteristic M1 and M2 macrophage expressed genes, using RT-PCR. For M1 macrophage markers, *Tnf*, *Nos2*, and *IL-12a* were assessed and for M2 markers, *Arg1*, *Retnla*, and *Chi313* were assessed by quantitative RT-PCR (S1 Table and Fig 1).

### Western blot analysis

Cell lysis was performed with 3-((3-cholamidopropyl) dimethylammonio)-1-propanesulfonic (CHAPS) acid buffer. Western blot analysis was performed using primary antibodies: polyclonal rabbit anti-APOL1 (HPA018885, 1:500, Sigma, St. Louis, MO), mouse monoclonal anti-GAPDH (CB1001, 1:10,000, Darmstadt, Germany), and secondary antibodies anti-rabbit IgG HRP (W401B, 1:10,000; Promega, Madison, WI) and anti-mouse IgG HRP (W402B, 1:10,000; Promega, Madison, WI).

### Flow cytometry

For evaluate whether bone marrow-derived macrophages were correctly differentiated, 9 d-cultured cells were stained with anti-macrophage antibodies; PE conjugated anti-F4/80 (BD Bioscience, San Jose, CA) and FITC conjugated anti-CD11b (BioLegend, San Diego, CA). Almost all of the BM-derived cells after 9 days of differentiation were F4/80+CD11b+. (Fig 1A and 1B). A FACScan flow cytometer (BD Biosciences, San Jose, CA) was used to acquire 10,000 events for each sample.

### Gene expression of lipid-related genes in the NEPTUNE study

Glomerular gene expression data from subjects with primary glomerular disease was obtained fron the Nephrotic Syndrome Study Network (NEPTUNE) [7]. Glomerular gene expression profiling was performed using Human Genome ST 2.1 Affymetrix Gene Chip arrays and processed using Affymetrix Power Tools. The robust multichip averaged (RMA) and normalized log2-transformed datasets were then batch corrected using ComBat (GenePattern, Broad Institute, Boston, MA and University of San Diego, San Diego, CA). Differential expression analysis between the controls and relevant disease groups was performed using Significance Analysis of Microarray (SAM) as implemented in the TIGR Multiexperiment Viewer software suite [8]. Transcripts passing an false discovery rate (FDR) correction (q value) for multiple testing below 5% were considered significantly differentially regulated. The gene expression data set was used to define co-expression of lipid related genes with *APOL1* in a genotype specific manner. Different sets of lipid related gene transcripts correlated with *APOL1* expression in patients carrying zero *APOL1* risk alleles and in those carrying two *APOL1* risk alleles.

### Human subjects research approvals

Subjects were recruited for an observational clinical protocol (NEPTUNE) at over 20 particpating sites; approvals were obtained in advance from institutional review boards. Approval for the particular studies described here were obtained from the NIDDK Intramural Research program Institutional Review Board, Bethesda, MD.

### Statistical analysis

Data were analyzed using Mann-Whitney U test to determine significant differences between two groups. A one-way analysis of variance (ANOVA) followed by Turkey’s multiple comparison test was performed to see significant differences among groups. The *P* value < 0.05 was considered as statistical significance. Values are represented as median and IQR. Statistical analyses were performed using GraphPad Prism software program (San Diego, CA).

## Results

### Characterization of the BAC/APOL1 mouse lines

*APOL1* transgene mRNA was expressed in kidney, liver and spleen and in macrophages isolated from bone marrow (S2 Fig). APOL1 protein was present in serum from the transgenic mice (S3 Fig). The mice had no clinical or pathologic phenotype when studied to at least 6 months of age.

### Cholesterol accumulation in kidney and spleen from APOL1-G1 or -G2 risk variant mice

**Fig 2:**
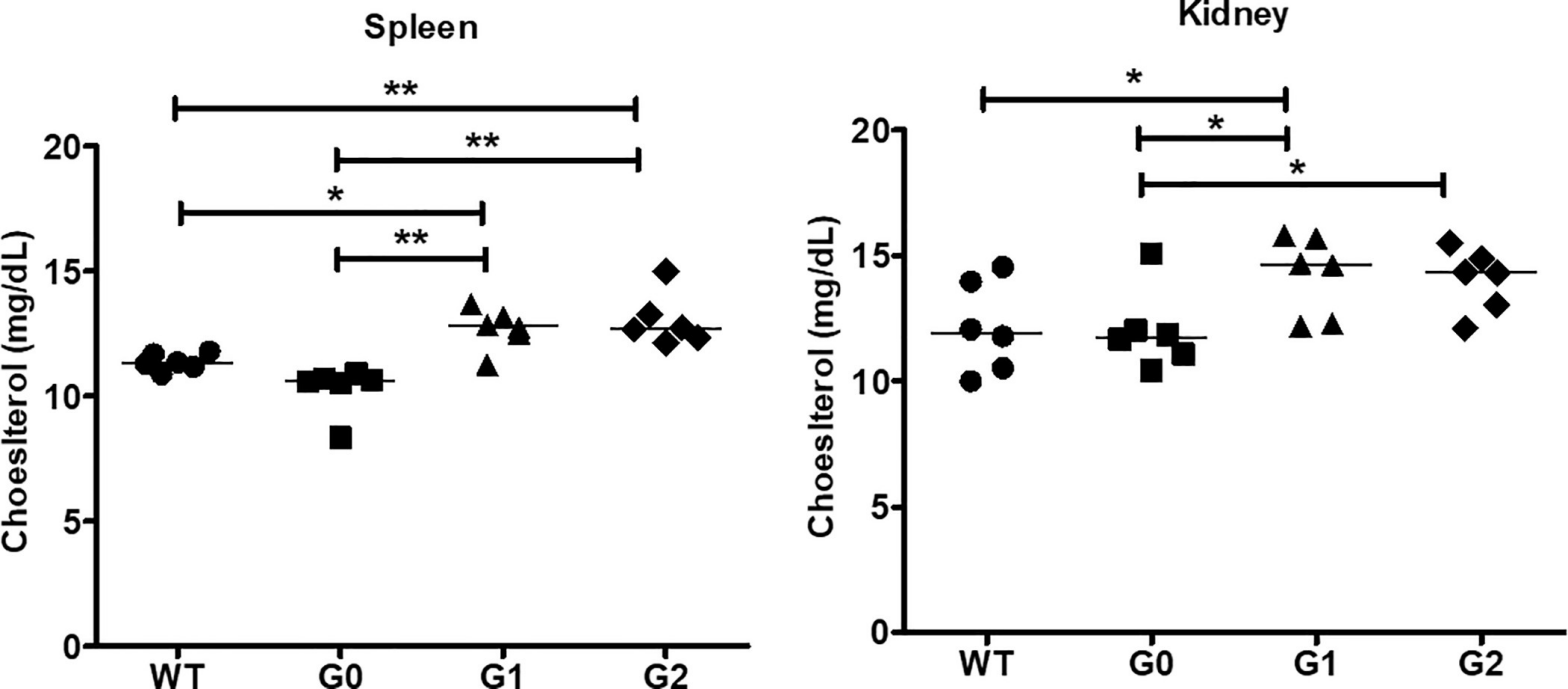
Cholesterol accumulation in BAC/APOL1 murine kidney and spleen. Total tissue cholesterol was increased in APOL1-G1 and APOL1-G2 mouse kidney and spleen compared to APOL1-G0 mouse kidneys, while APOL-G0 tissue cholesterol was similar to wild type (WT). Each data point represents of individual animal, with the value representing the average of duplicate measurements (N = 4 mice samples for each group). WT; wild type. * *P* < 0.05.

Total cholesterol content was higher in kidney and spleen from APOL1-G1 and -G2 mice compared to APOL1-G0 mice. By contrast, cholesterol content in kidney and spleen was similar between APOL1-G0 and WT mice (Fig 2). Semi-quantitative scoring of Oil red O stained sections demonstrated increased glomerular lipid among all three *APOL1* transgenic lines, particularly following renal injury, whereas tubular lipids were less markedly and consistently affected (S7 Fig). These data suggest that APOL1 renal risk variants perturb cellular cholesterol homeostsasis.

### ABCA1 and ABCG1 mRNAs expression in BAC/APOL1 kidney and spleen

**Fig 3:**
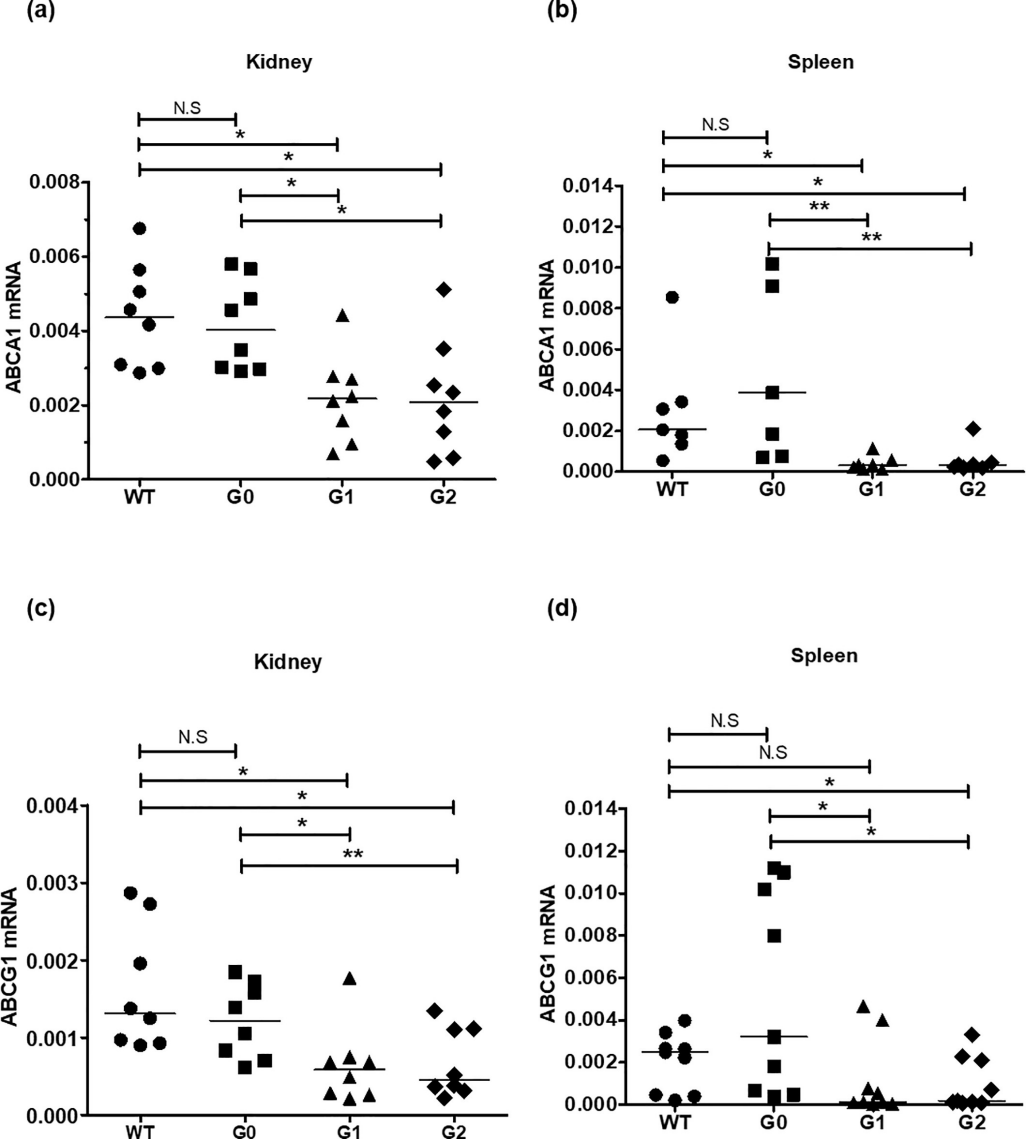
Expression of cholesterol transporters in BAC/APOL1 mouse kidney and spleen. Total RNA was isolated from kidney and spleen, and relative ABCA1 and ABCG1 mRNA levels were evaluated by quantitative RT-PCR. ABCA1 mRNA levels were lower in APOL1-G1 and APOL1-G2 risk variant (A) kidney and (B) spleen compared to APOL-G0 mice. ABCG1 mRNA levels were also lower in the APOL1-G1 and APOL1-G2 groups of (C) kidney and (D) spleen compared to APOL1-G0 mice. WT; wild type. Median is shown by a horizontal bar. Mann-Whitney U-test; * *P* < 0.05, ** *P* < 0.01.

ABCA1 and ABCG1 are the major transcellular membrane cholesterol transporters and key regulators of cell cholesterol homeostasis. To assess a possible association between cholesterol accumulation and expression of membrane cholesterol transporters, we performed quantitative RT-PCR about ABCA1 and ABCG1 genes in kidney and spleen. Interestingly, ABCA1 mRNA levels were down-regulated in both kidney and spleen of APOL1-G1 and -G2 compared with WT or APOL1-G0 (**Fig 3A and 3B**). ABCG1 mRNA levels were also decreased in APOL1-G1 and -G2 compared to WT or APOL1-G0 mice (Fig 2C and 2D). By contrast, cholesterol transporter mRNA levels were similar between APOL1-GO mice and WT mice.

### Intracellular cholesterol accumulation in M1 and M2 macrophages in BAC/APOL1 mice

**Fig 4:**
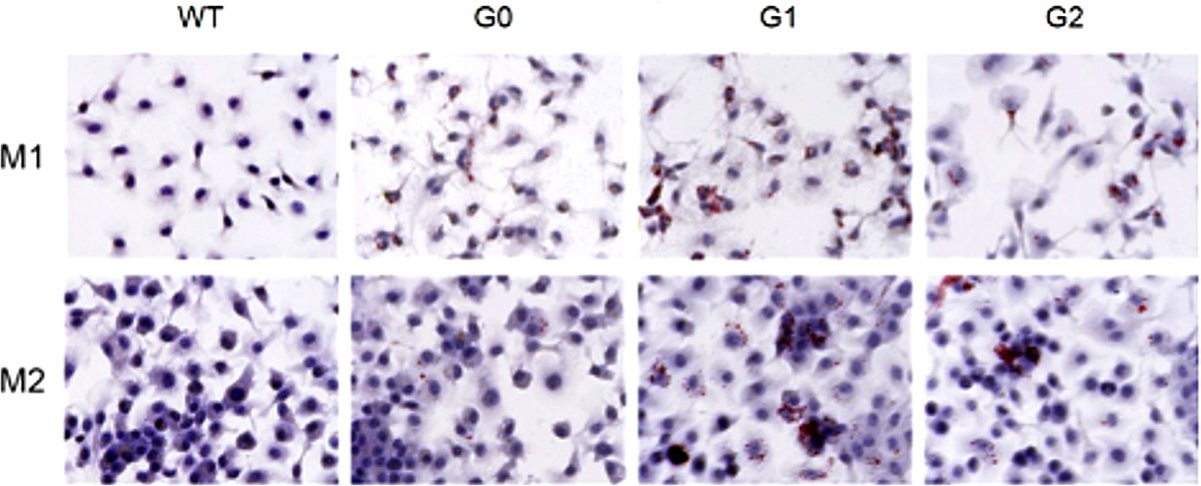
Cholesterol accumulation in BAC/APOL1 mouse bone marrow (BM)-derived macrophages. BM-derived macrophages were polarized into M1 or M2 macrophages by of IFN-γ (20 ng/mL) or IL-4 (20 ng/mL) respectively for 48 h and total cholesterol levels were measured. BM-derived macrophages were polarized into M1 or M2 macrophages. For M1, IFN-γ (20 ng/mL) and for M2, IL-4 (20 ng/mL) was treated for 48 h. To develop lipid-laden macrophages, cells were incubated with aggregated LDL (50 μg/mL) for 24 h. Cells fixed with 10% formalin, then stained with Oil Red O (Original magnification, X400). (A) In M1 macrophages, cholesterol levels were higher in APOL1-G1 and APOL1-G2 mice compared to both WT and APOL1-G0 mice. (B) M2 macrophages exhibited similar finidngs, but APOL1-G2 macrophages did not show enhanced cholesterol content. * *P* < 0.05, ** *P* < 0.01.

Macrophages play an important role in cholesterol homeostasis in peripheral, extra-hepatic tissues. Therefore, we examined intracellular cholesterol content in M1 and M2 macrophages following loading with LDL. M1 macrophges with APOL1-G1 and -G2 variants showed increased total cellular cholesterol compared to WT or APOL1-G0 cells (Fig 3). By contrast, cholesterol loading only increased in APOL1-G1 M2 macrophages increased cholesterol quantity compared to WT or APOL1-G0, and APOL1-G2 macrophages were similar to control (**Fig 3A**). Oil red O stain of macrophages confirmed this pattern with prominent and numerous intracellular lipid droplets in APOL1-G1 and -G2 cells compared to WT or APOL1-G0 macrophages, with regard to both M1 and M2 macrophages (**Fig 4**). These findings suggest a direct effect of APOL1-G1 and -G2 risk variant expression on intracellular cholesterol content.

### [^3^H]-cholesterol efflux from M1 and M2 macrophages

**Fig 5:**
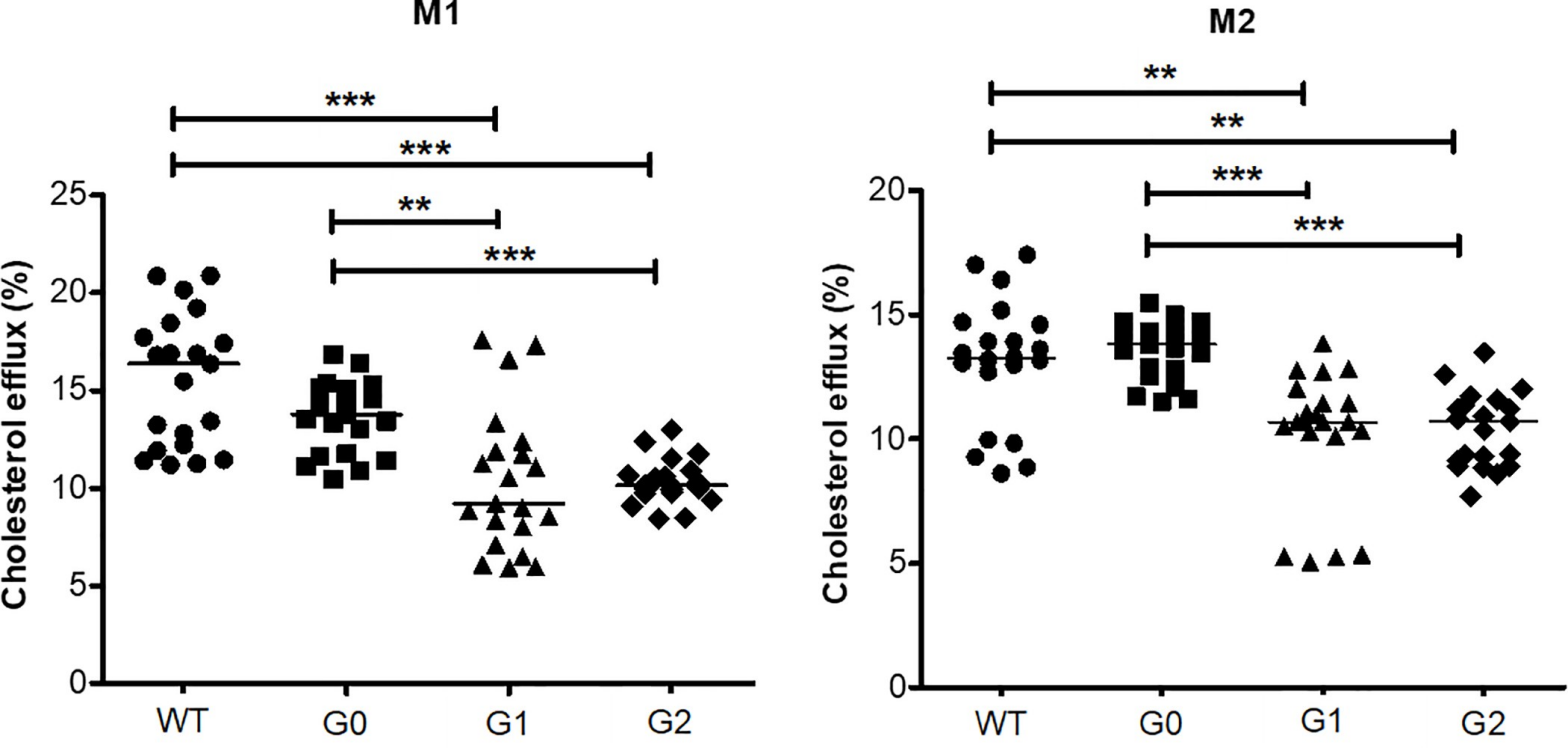
[^3^H]-Cholesterol efflux from BAC/APOL1 mouse bone marrow-derived macrophages. Cultured macrophages were polarized into M1 macrophages with IFN-γ (20 ng/mL) and M2 macrophages with IL-4 (20 ng/mL). Following labeling macrophages with [^3^H]-cholesterol during 18 h, each [^3^H]-cholesterol efflux into media was induced by 4 h-incubation with the mouse serum as a cholesterol acceptor. Cholesterol efflux was reduced in APOL1-G1 and APOL1-G2 macrophages compared to APOL1-G0 macrophages;.this was true for both M1 and M2 macrophages. The results are representative of independent experiment (N = 6 mice per group, 3–5 samples per mouse; median is shown). One-way ANOVA test; **P < 0.01, ***P < 0.001.

To evaluate whether APOL1 risk variants are associated with reverse cholesterol transport macrophage, we measured [^3^H]-cholesterol efflux capacity in M1 and M2 macrophages (**Fig 5)**. Cholesterol efflux rates were significantly decreased in M1 macrophages carrying G1 and G2 variants compared to WT or G0 variant, with no difference between WT and APOL1-G0 macrophages (16.37 ± 6.03% in WT; 13.79 ± 3.42% in G0; 9.22 ± 4.60% in G1; 10.14 ± 1.01% in G2, *P* < 0.001). Similarly, in M2 macrophages, cholesterol efflux was reduced in the macrophages expressing APOL1-G1 or -G2 variants compared to WT or G0 (13.28 ± 3.32% in WT; 13.81 ± 1.76% in G0; 10.68 ± 1.54% in G1; 10.69 ± 2.45% in G2, *P* < 0.001). Thus, cholesterol export was reduced in both M1 and M2 macrophages in the presence of the APOL1 G1 and G2 alleles.

### ABCA1 and ABCG1 mRNA expression in BAC/APOL1 macrophages

**Fig 6:**
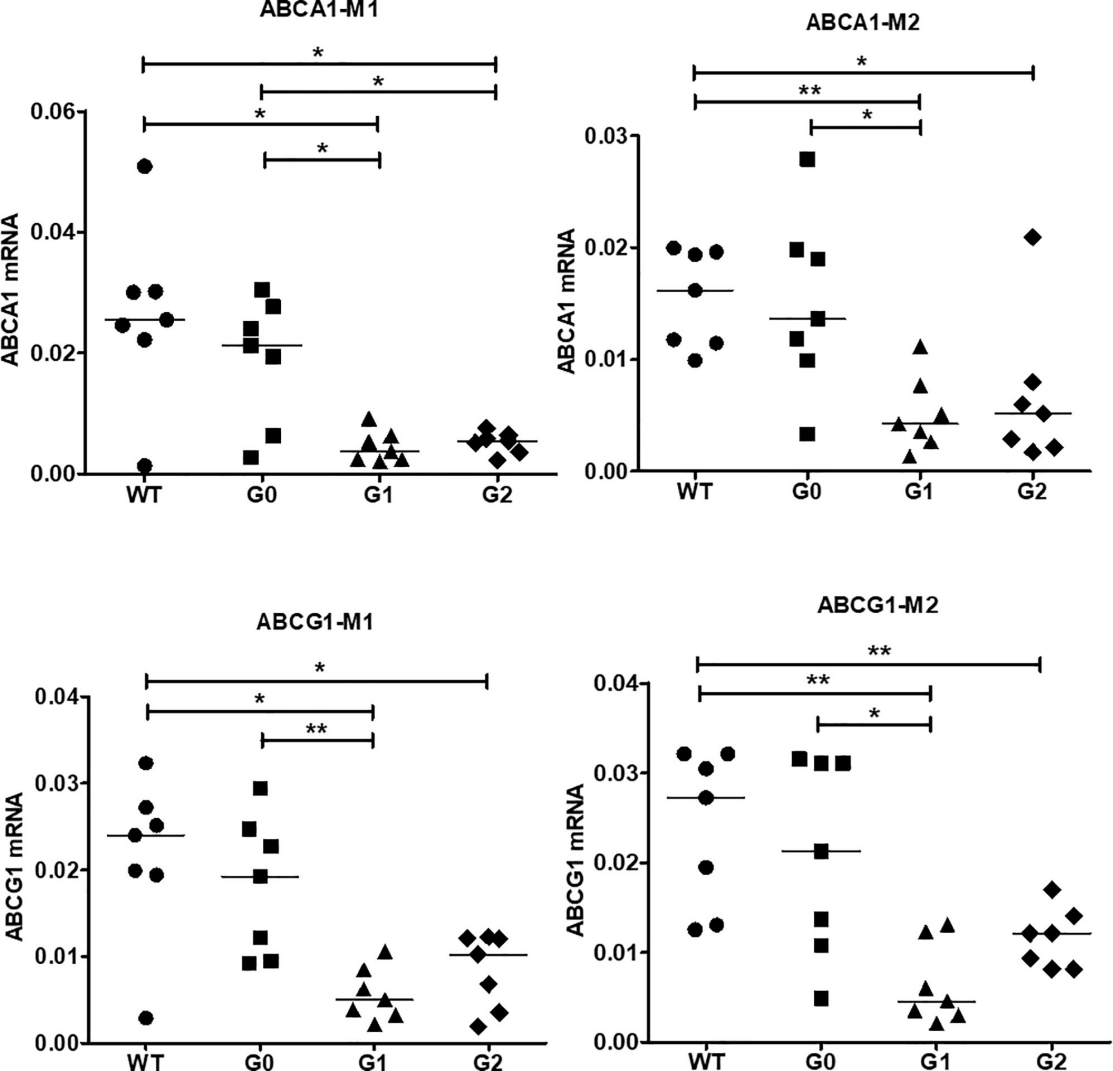
ABCA1 and ABCG1 mRNA expression in BAC/APOL1 mouse BM-derived macrophages. ABCA1 and ABG1 RNA was reduced in APOL1-G1 compared to APOL1-G0 M1 macrophages, whereas for APOL1-G2 macrophages only M1 macrophages showed a significant reduction. Mann-Whitney U-test; * *P* < 0.05, ** *P* < 0.01.

We next evaluated mRNA expression of the cholesterol transporter genes, *ABCA1* and *ABCG1* in M1 and M2 macrophages. Both ABCA1 and ABCG1 mRNA levels were significantly decreased in APOL1-G1 and -G2 variants compared to WT or APOL1-G0 in M1 and even M2 macrophages (**Fig 6**) suggesting a direct effect of APOL1 risk variant expression on the expression of ABC transporters, ABCA1 and ABCG1.

### APOL1-mediated intracellular cholesterol accumulation is independent of ABC transporters mediated efflux in HeLa cells

**Fig 7:**
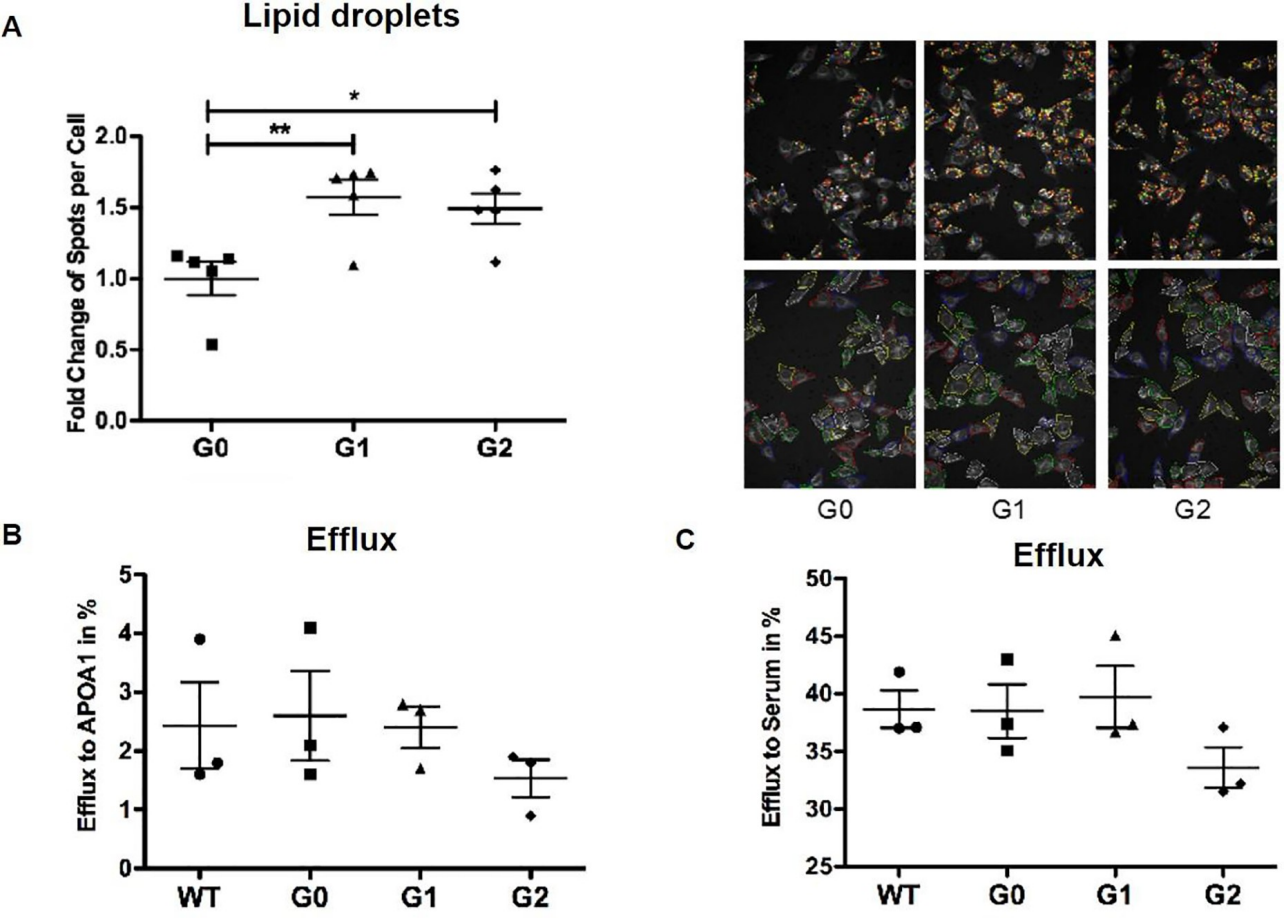
APOL1 risk variant expression in HeLa cells leads to lipid accumulation in the absence of decreased cholesterol efflux. HeLa cells were infected with different APOL1 alleles using a lentiviral system. **(A)** HeLa cells were fixed and stained with BODIPY 493/503 and cell-mask blue. The number of lipid droplets per cell was determined by high throughput Perkin Elmer OPERA analysis and Acapella Image Analysis software. Dot blot analysis indicating a significantly increased number of Bodipy493/503-positive lipid droplets per cell in HeLa cells infected with APOL1 G1 or APOL1 G2 risk variants when compared to APOL1 G0 transfected HeLa cells (left). Representative images of Bodipy493/503 staining (right, upper panel) to identify lipid droplets and cell-mask staining (right, lower panel) to identify individual cells. *p<0.05, **p<0.01. **(B,C)** HeLa cells expressing the different *APOL1* alleles were labeled with 1 μCi/ml ^3^H-cholesterol in medium with 1% FBS for 24 hours, then washed with PBS, incubated in RPMI containing 2% human serum for 6 hours. Dot plot analysis indicating no changes in cholesterol efflux to APOA1 **(B)** or human serum **(C)** in APOL1 G1 and APOL1 G2 risk variant expressing HeLa compared to APOL1 G0 expressing cells. Both APOL1-G1 and APOL1-G2 HeLa cells showed increased lipid droplets compard to APOL1-G0 HeLa cells. There were no statistically significant differences in cholesterol efflux that were detected in this system.

To further investigate the mechanism by which APOL1 risk variant expression affects the cellular cholesterol content, HeLa cells, which do not express ABCA1 and ABCG1 as previously shown [9, 10] were stably infected with lentivirus carrying the *APOL1* risk variants under the CMV promoter. APOL1 expression levels were determined by Western blot and quantitative real-time PCR analysis (S8 Fig). Whereas uninfected HeLa cells show very low levels of endogenous APOL1 expression, APOL1 expression was significantly increased when HeLa cells were infected with APOL1 G0, G1 and G2. Interestingly, APOL1 expression was significantly higher in APOL1 G0 infected HeLa cells when compared to G1 and G2 infected cells. Cellular cholesterol content was determined using BODIPY staining followed by high throughput Perkin Elmer OPERA analysis. We found that APOL1 G1 and G2 expressing HeLa cells show significantly increased lipid droplet content when compared to APOL1 G0 expressing HeLa cells (**Fig 7A)** in the absence of ABC transporter mediated changes in cholesterol efflux (**Fig 7B and 7C**). This suggests that APOL1 risk variant expression may affect intracellular cholesterol content in an ABCA1/ABCG1 independent manner.

### Clinical study: NEPTUNE

In order to understand the role of *APOL1* risk variants. we studied the association of glomerular APOL1 expression with lipid related gene expression. Different sets of lipid related gene transcripts correlated with ApoL1 expression in patients carrying zero versus *APOL1* two risk alleles (S2 Table). In particular, several genes involved in cholesterol efflux were positively correlated with *APOL1* mRNA expression in patients carrying two *APOL1* risk alleles (n = 15, P<0.05, |r| = 0.52–0.62) while *APOL1* mRNA expression negatively correlated with cholesterol and lipid associated transcript levels in patients with zero *APOL1* risk alleles (n = 17,P<0.05, |r| = 0.52–0.67). While these data do not provide a direct biological explanation for the mechanisms of APOL1 action with regard to lipid homeostasis, the correlated expression does sugget that *APOL1* may interacts with other genesin a genotype specific manner.

## Discussion

In this study, we showed that APOL1-G1 and -G2 genetic variants are associated increased intracellular cholesterol content, with ABCA1 and ABCG1 repression and with decreased reverse cholesterol transport via suppressed ATP-binding cassette transporter gene expression (ABCA1 and ABCG1). These data were consistent in cultured macrophages and liver and spleen from transgenic mice. The data suggest that impaired cholesterol efflux capacity from APOL1 renal risk variant macrophages may promote formation of foam cells in vivo. Our study showed peripheral cholesterol accumulation was similarly increased in both M1 and M2 macrophages from APOL1-G1 and -G2 genetic variants although this finding may be confounded to some extent by reduced expression of the renal risk variants compared to the common G0 allele (S8 Fig) or by toxicity induced by the renal risk variants. In general, M1 macrophages to play a role in host defense against various pathogens, and are involved in inflammatory responses. In contrast, M2 macrophages have anti-inflammatory and/or anti-fibrotic function [3, 11, 12].

APOL1 is a minor protein constituent of plasma HDL and is lipophilic, partly explaining its localization to HDL. APOL1 functions in HDL include anti-trypanosomal activity [13, 14]; other functions are possible but have not been explored. APOL1 is also expressed intracellularly in various cells, include hepatocytes, podocytes and endothelial cells. However, the physiologic function of intracellular APOL1 is not well known. Although APOL1 is a component of the HDL particle (and specifically the HDL3 particle) [15–17], APOL1 plasma level is not directly correlated with serum HDL levels [16] but are positively associated with total cholesterol and triglycerides levels, as well as glucose [16, 18]. In familial hypercholesterolemia, APOL1 serum levels are reduced and lower levels predict adverse cardiac events [19]. Furthermore, the APOL1 renal risk variants are associated lower plasma concentrations of medium-sized HDL, in a stepwise fashion (effect of 2 > 1 > 0), and it has been proposed that the APOL1 variants reduce production of medium-sized HDL [20]. Medium and small HDL particles are the species most protective against cardiovascular disease [21]. These findings suggest the hypothesis that APOL1 renal risk variants might alter lipid homeostasis in a cardioprotective manner, perhaps by affecting cholesterol transport in peripheral tissues. In clinical studies, data on associations of the APOL1 renal risk variants with cardiovascular disease have been complex, with evidence for increased risk [22, 23] and also with decreased carotid artery calcified plaque and improved survival [24].

In conclusion, the present study suggests a pathophysiologic relationship between *APOL1* genetic risk variants and impaired reverse cholesterol transport through decreased expression of cholesterol efflux transporters in peripheral tissues. This process would tend to promote macrophage foam cell formation, driving inflammation in the glomerulus and renal interstitium. This hypothesis offers a possible mechanism contributing to renal and vascular injury characteristic of the *APOL1* renal risk variants.

## Supporting information

S1 Fig Transgene construct. Shown is the 48 kb transgene construct that was used to generate the BAC/APOL1 transgenic mice. The 6 coding exons of APOL1 are numbered. Exon 6 contains the G1 and G2 renal risk variants. The brown arrows show excision sites that were engineered to allow deletion of exon 6, an approach not used in the mice described here. Exons of the flanking genes are shown in turquoise, *APOL2* on the left and *MYH9* on the right.
(DOCX)

S2 Fig Transgene protein expression. Shown is a Western blot for APOL 1, which appears as a dimer located at approximately 42 and 44 kDa. Sera (2 μl) from the following mice were run on the gel: wild-type mouse as a negative control; BAC APOL1-G0 mouse,BAC APOL1-G1 mouse, BAC APOL1-G2 mouse and as a positive control, mice expressing APOL1-G0 under the conrtrol of an albumin promoter. APOL1 expression levels are similar among the strains.
(PDF)

S3 Fig Transgene RNA expression. RNA was extracted from macrophage and tissues using the Ribozol reagent according to the manufacture’s instructions. RNA isolation was performed using RNA isolation kit (Machery-Nagel, Bethlehem, PA, USA). Complementary DNA was synthesized using the GoScript reverse transcriptase system (Promega, Madison, WI, USA). PCR was performed for APOL1 and the primer sequence was like the follows; Forward, CAATGTGGTGCTTGGCTCTCTC; Reverse, AATGCCTCGTGTTGAGTTGGTAAG.
(PPTX)

S4 Fig Immunostaining of APOL1 in glomeruli. Frozen sections of mouse kidney were subjected indirect immunofluorescence staining using antibodies directed against APOL1 (green) and podocalyxin (red). Podocalyxin antibody was from R&D Biosystems.
(PPTX)

S5 Fig APOL1 expression in transgenic mouse liver. APOL1 expression was highest around the hepatic venules, with expression levels APOL1 G0 > G1 > G2. Mouse APOL1 genotpypes are shown; "wild" denotes wild-type.
(PPTX)

S6 Fig APOL1 expression in transgenic mouse lung. Minimal expression is seen. Mouse APOL1 genotpypes are shown.
(PPTX)

S7 Fig Glomerular and tubular lipids in the SAND model of renal inury. Litter mate control (LC) or APOL1 mice with genotypes as shown were left intact (control) or were challenged to induced kidney injury with SAND (saline for drinking), Angiotensin II subcutaneous pump, nephrectomy, and 11-deoxycorticosterone subcutaneous pellets). Data are summarized as median and IQR.
(PPTX)

S8 Fig Characterization of Hela cells transduced with APOL1 expressoin constructs. Representative western blot analysis (left) and quantitative real-time PCR analysis (right) of APOL1 expression in HeLa cells transduced with different APOL1 alleles (G0, G1, G2).
(DOCX)

S1 Table Primers for evaluation of macrophage polarization markers. (DOCX)

S2 Table Expression of lipid-related genes correlates with APOL1 APOL1 G0 and G2 RNA expression in kidney biopsies from the NEPTUNE study. Both direct assocations (higher APOL1 gene expression, higher gene expression) and indirect associations (higher APOL1 expression, lower gene expression) are shown.
(DOCX)

S1 Data The individual data points are presented for S8 Fig, expression of APOL1 RNA normalized to expression of GAPdH. (XLSX)
