# Supplementary figures and images for: APOL1 renal risk variants promote cholesterol accumulation in tissues and cultured macrophages from APOL1 transgenic mice
Source: PLoS One. 2019 Apr 18;14(4):e0211559. doi: 10.1371/journal.pone.0211559 (PMC6472726; doi:10.1371/journal.pone.0211559)

**Supplemental Figure 1. Transgene construct.**


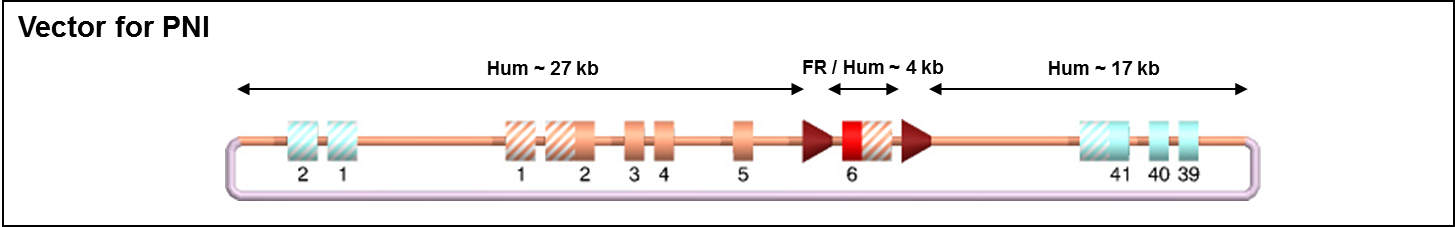

Supplement: S1 Fig — Shown is the 48 kb transgene construct that was used to generate the BAC/APOL1 transgenic mice. The 6 coding exons of APOL1 are numbered. Exon 6 contains the G1 and G2 renal risk variants. The brown arrows show excision sites that were engineered to allow deletion of exon 6, an approach not used in the mice described here. Exons of the flanking genes are shown in turquoise, APOL2 on the left and MYH9 on the right. (DOCX) [file pone.0211559.s001.docx]

## Slide 1
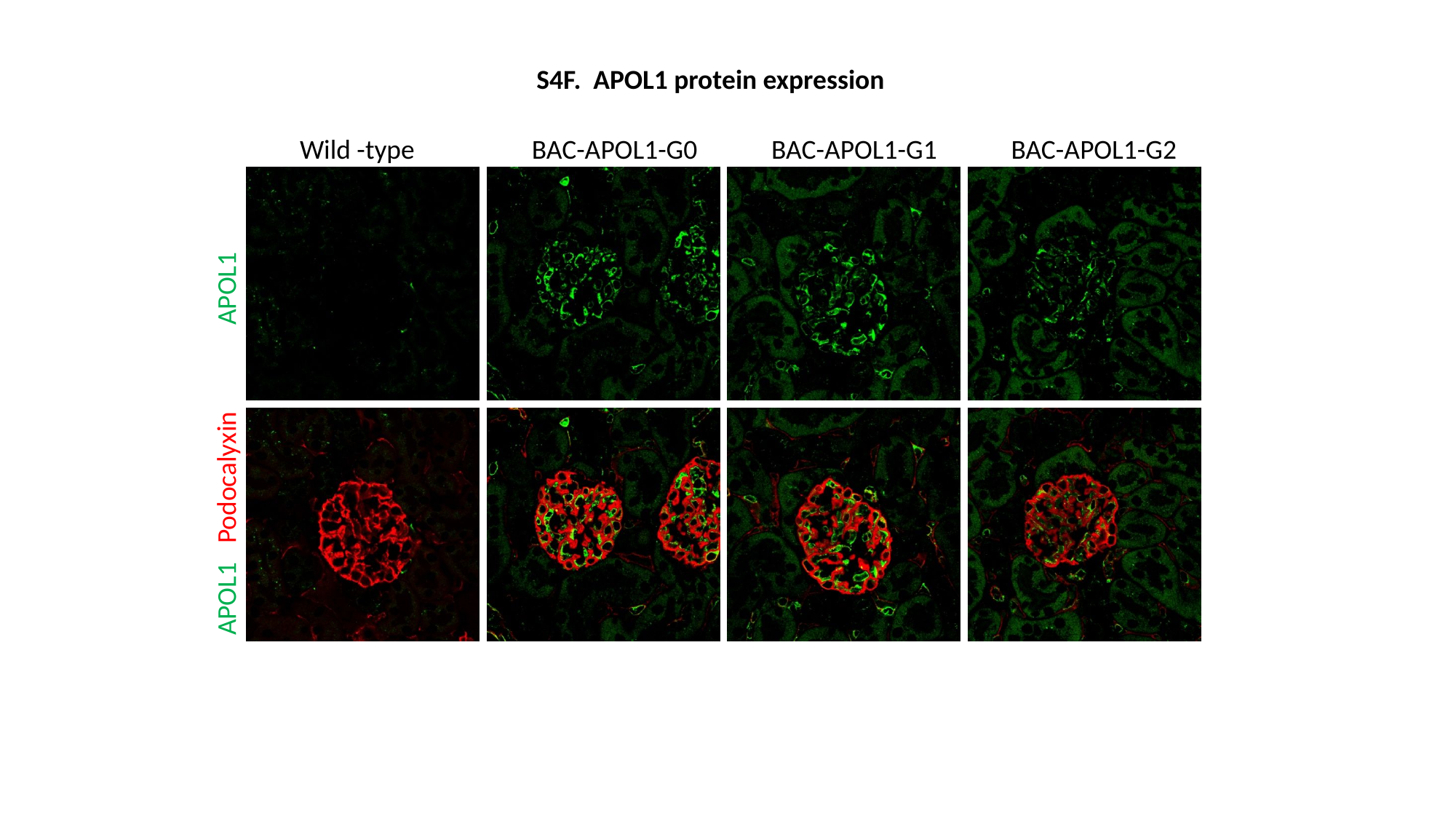

S4F. APOL1 protein expression
Wild -type BAC-APOL1-G0 BAC-APOL1-G1 BAC-APOL1-G2
APOL1 Podocalyxin APOL1

Supplement: S4 Fig — Frozen sections of mouse kidney were subjected indirect immunofluorescence staining using antibodies directed against APOL1 (green) and podocalyxin (red). Podocalyxin antibody was from R&D Biosystems. (PPTX) [file pone.0211559.s004.pptx]

## Slide 1
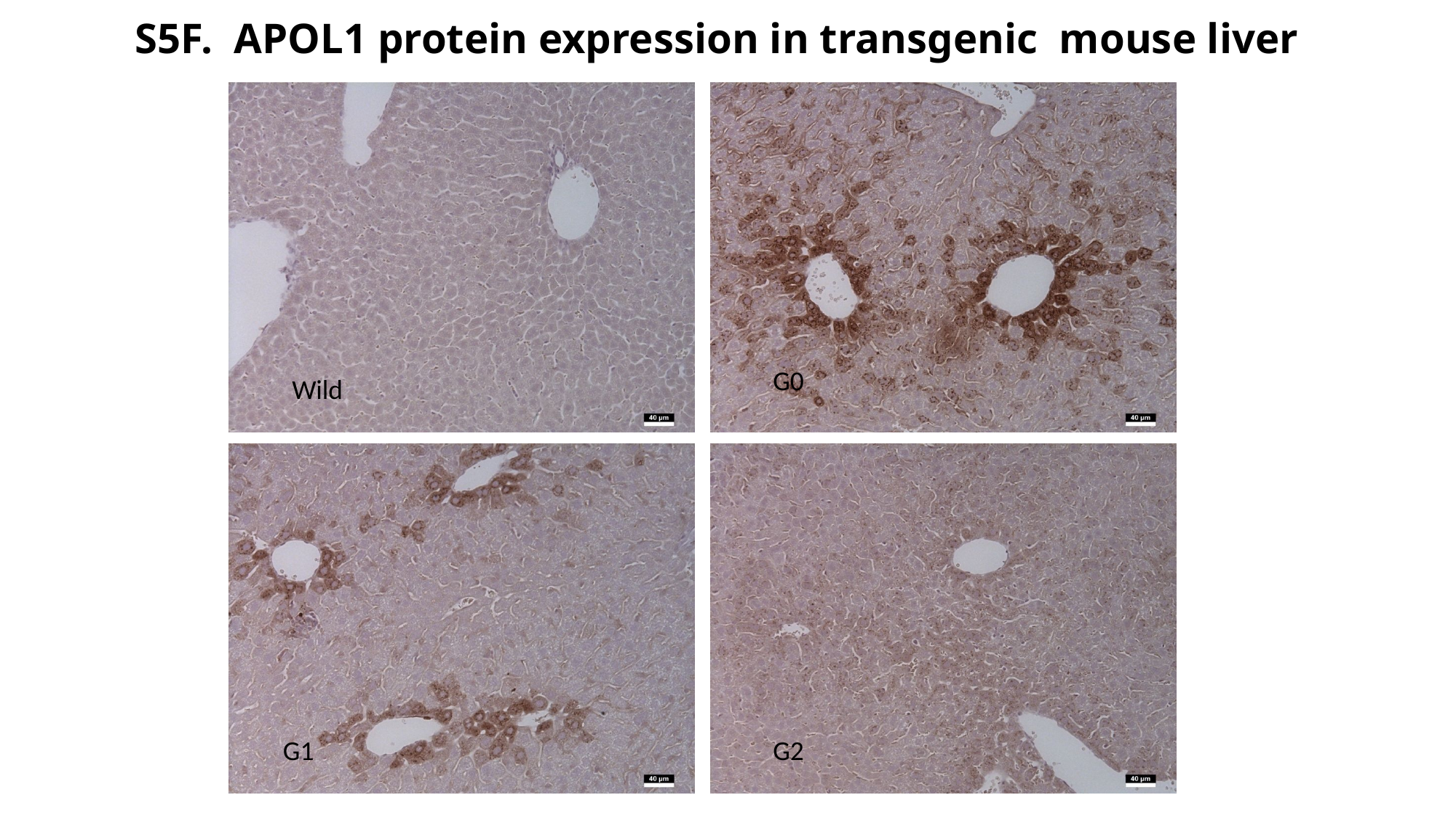

# S5F. APOL1 protein expression in transgenic mouse liver
G0
Wild
G1
G2

Supplement: S5 Fig — APOL1 expression was highest around the hepatic venules, with expression levels APOL1 G0 > G1 > G2. Mouse APOL1 genotpypes are shown; "wild" denotes wild-type. (PPTX) [file pone.0211559.s005.pptx]

## Slide 1
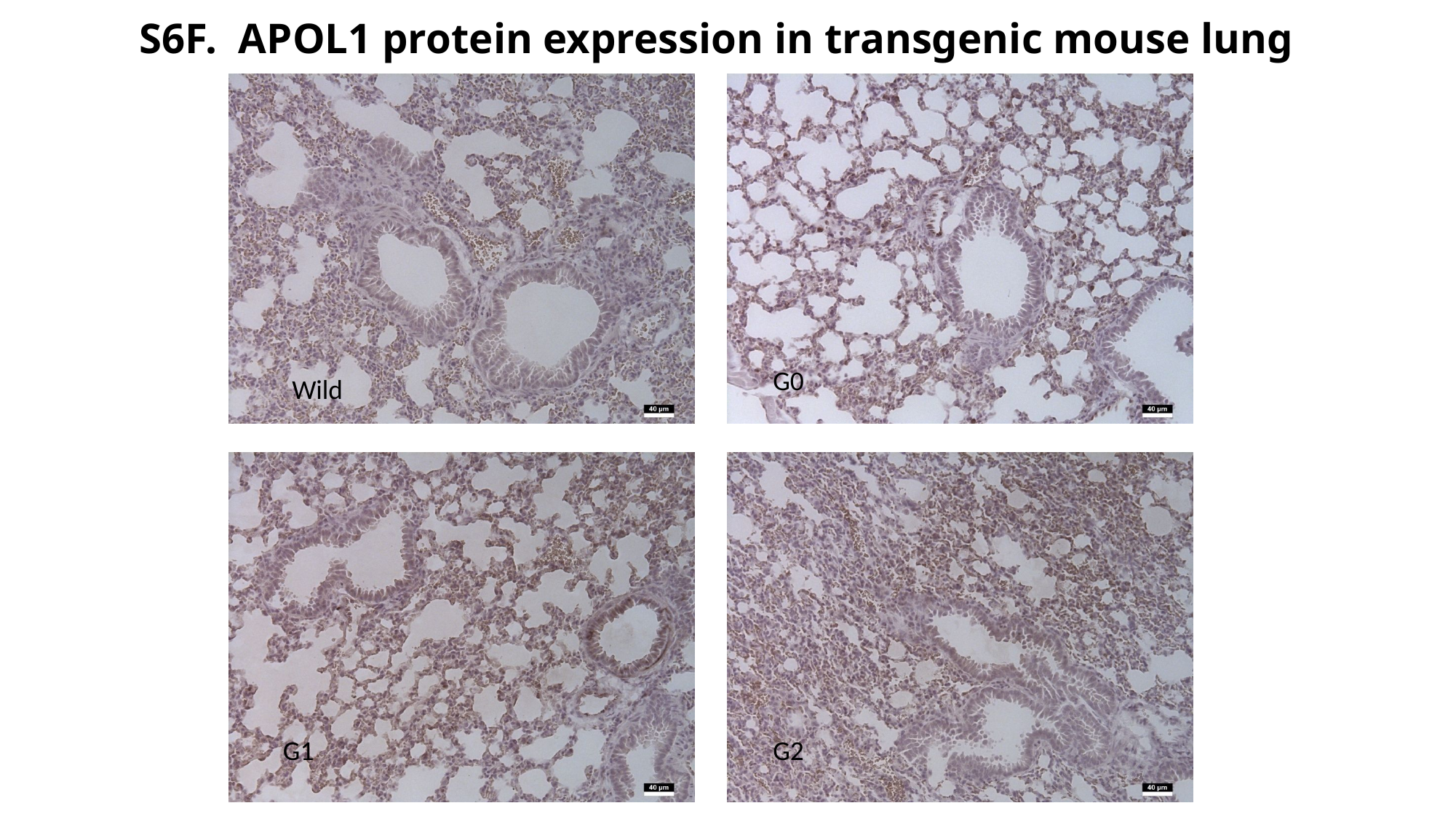

S6F. APOL1 protein expression in transgenic mouse lung
G0
Wild
G1
G2

Supplement: S6 Fig — Minimal expression is seen. Mouse APOL1 genotpypes are shown. (PPTX) [file pone.0211559.s006.pptx]

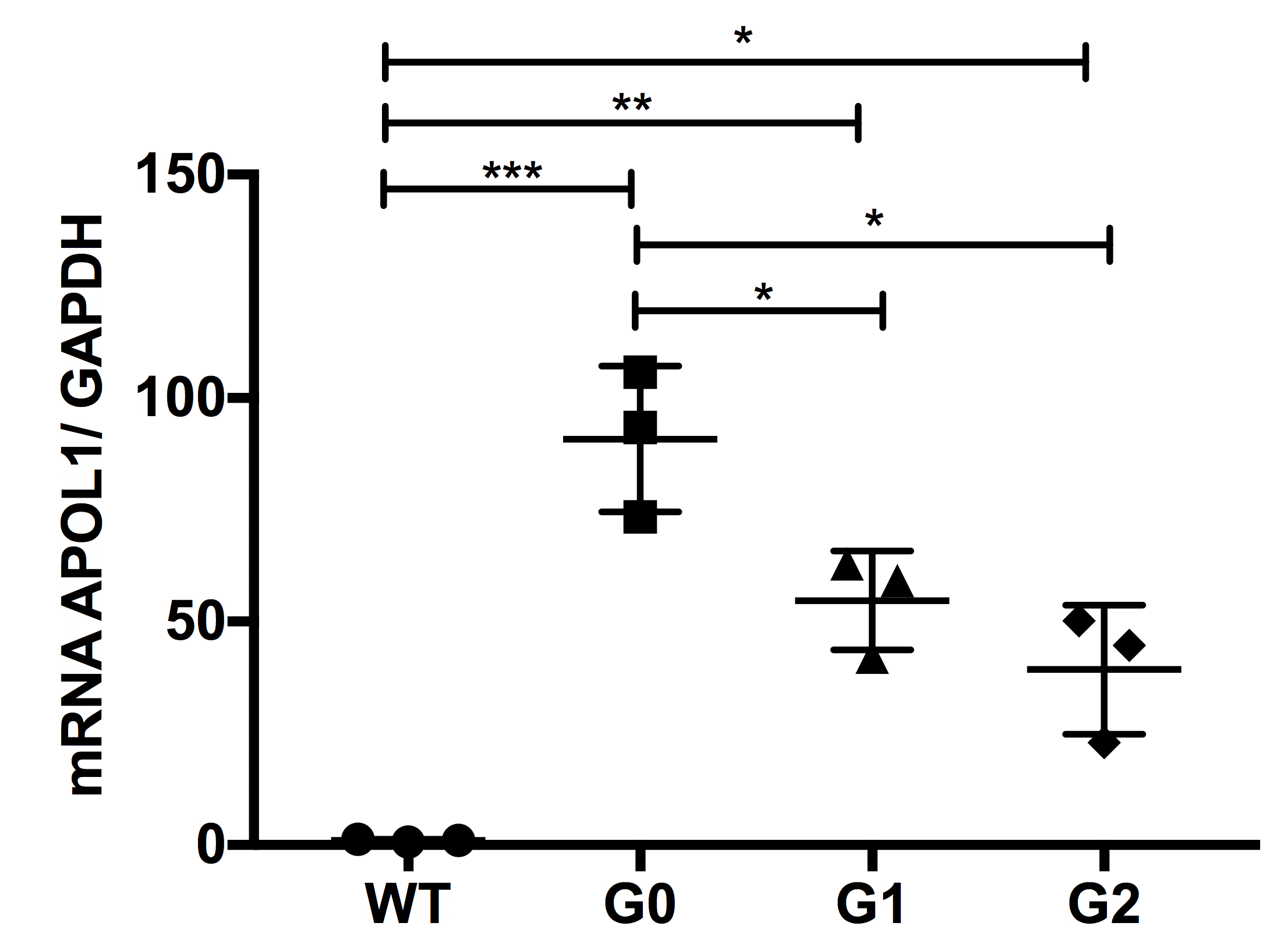
**Supplemental Figure 8. APOL1 protein and RNA expression in transfected HeLa cells.**


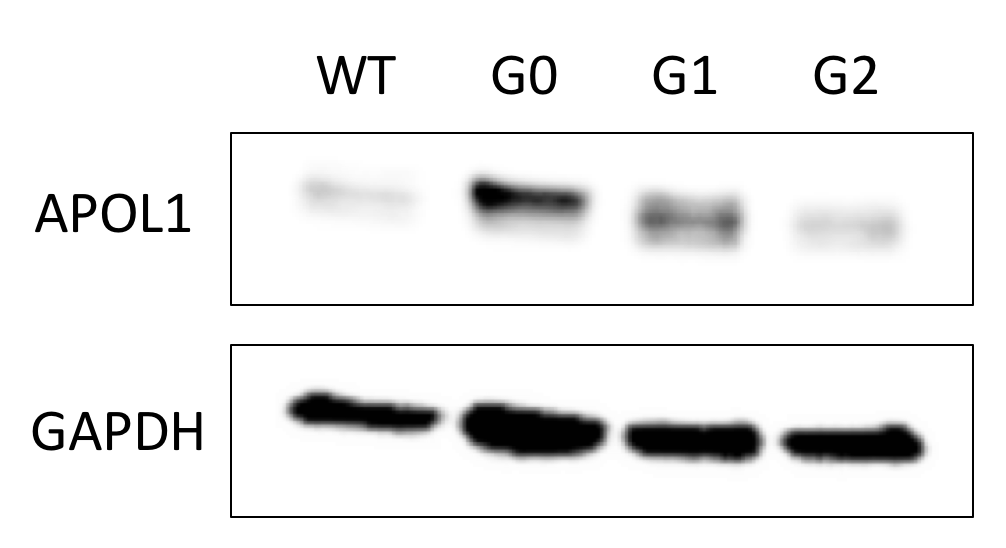

Supplement: S8 Fig — Representative western blot analysis (left) and quantitative real-time PCR analysis (right) of APOL1 expression in HeLa cells transduced with different APOL1 alleles (G0, G1, G2). (DOCX) [file pone.0211559.s008.docx]
