# Supplementary material for: APOL1 renal risk variants promote cholesterol accumulation in tissues and cultured macrophages from APOL1 transgenic mice
Source: PLoS One. 2019 Apr 18;14(4):e0211559. doi: 10.1371/journal.pone.0211559 (PMC6472726; doi:10.1371/journal.pone.0211559)
Supplement: S2 Fig — Shown is a Western blot for APOL 1, which appears as a dimer located at approximately 42 and 44 kDa. Sera (2 μl) from the following mice were run on the gel: wild-type mouse as a negative control; BAC APOL1-G0 mouse,BAC APOL1-G1 mouse, BAC APOL1-G2 mouse and as a positive control, mice expressing APOL1-G0 under the conrtrol of an albumin promoter. APOL1 expression levels are similar among the strains. (PDF) [file pone.0211559.s002.pdf]

Supplemental Figure 2

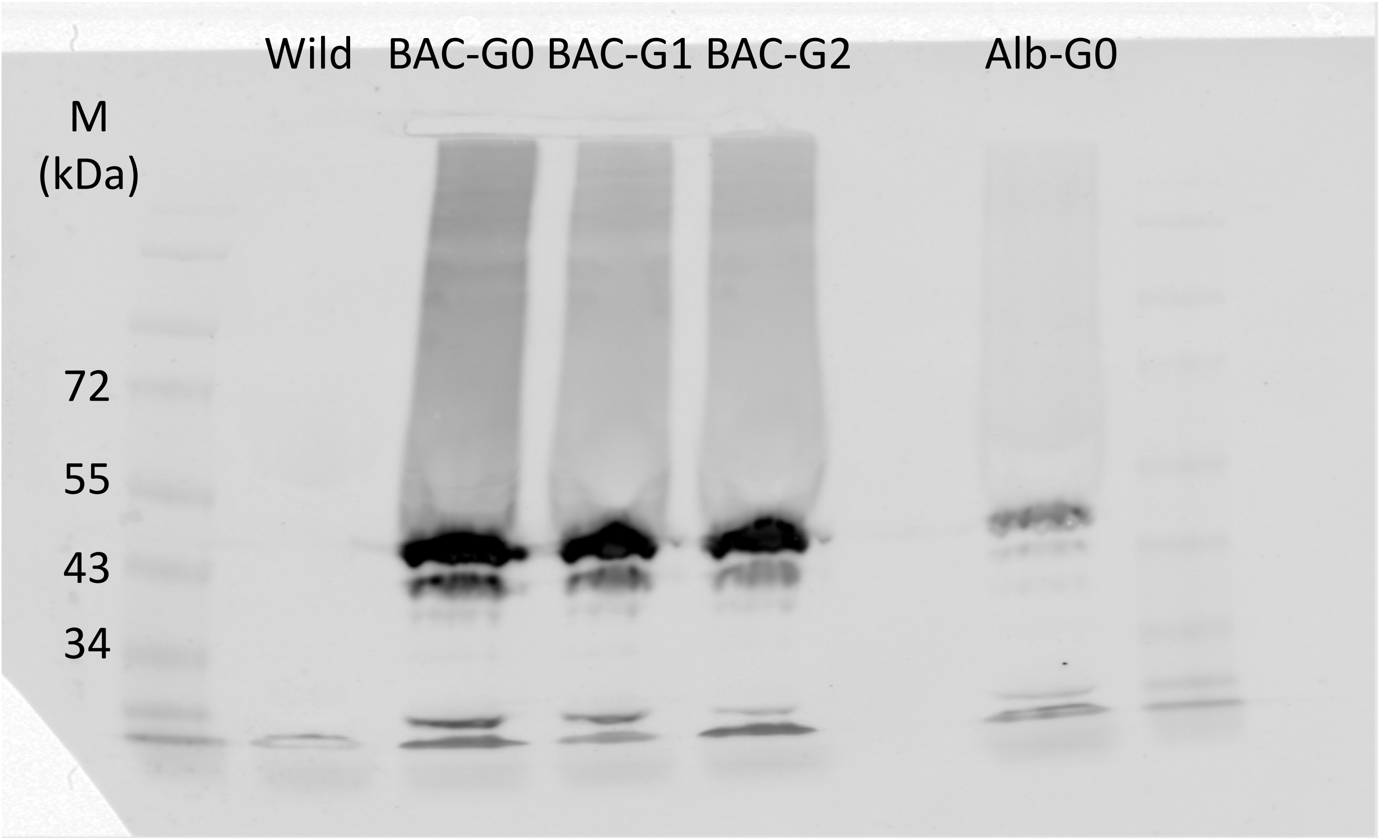

Legend. Shown is a Western blot for APOL1, which appears as a dimer located at approximately 42 and 44 kDa. Serum (2 µl) from the following mice were run on the gel: wild-type mouse (as a negative control), BAC APOL1-GO mouse, BAC APOL1-G1 mouse, BAC-APOL1G2 mouse and as a positive control, mouse expressing APOL1-G0 under the control of an albumin promoter.
