# Supplementary material for: APOL1 renal risk variants promote cholesterol accumulation in tissues and cultured macrophages from APOL1 transgenic mice
Source: PLoS One. 2019 Apr 18;14(4):e0211559. doi: 10.1371/journal.pone.0211559 (PMC6472726; doi:10.1371/journal.pone.0211559)
Supplement: S3 Fig — RNA was extracted from macrophage and tissues using the Ribozol reagent according to the manufacture’s instructions. RNA isolation was performed using RNA isolation kit (Machery-Nagel, Bethlehem, PA, USA). Complementary DNA was synthesized using the GoScript reverse transcriptase system (Promega, Madison, WI, USA). PCR was performed for APOL1 and the primer sequence was like the follows; Forward, CAATGTGGTGCTTGGCTCTCTC; Reverse, AATGCCTCGTGTTGAGTTGGTAAG. (PPTX) [file pone.0211559.s003.pptx]

## Slide 1
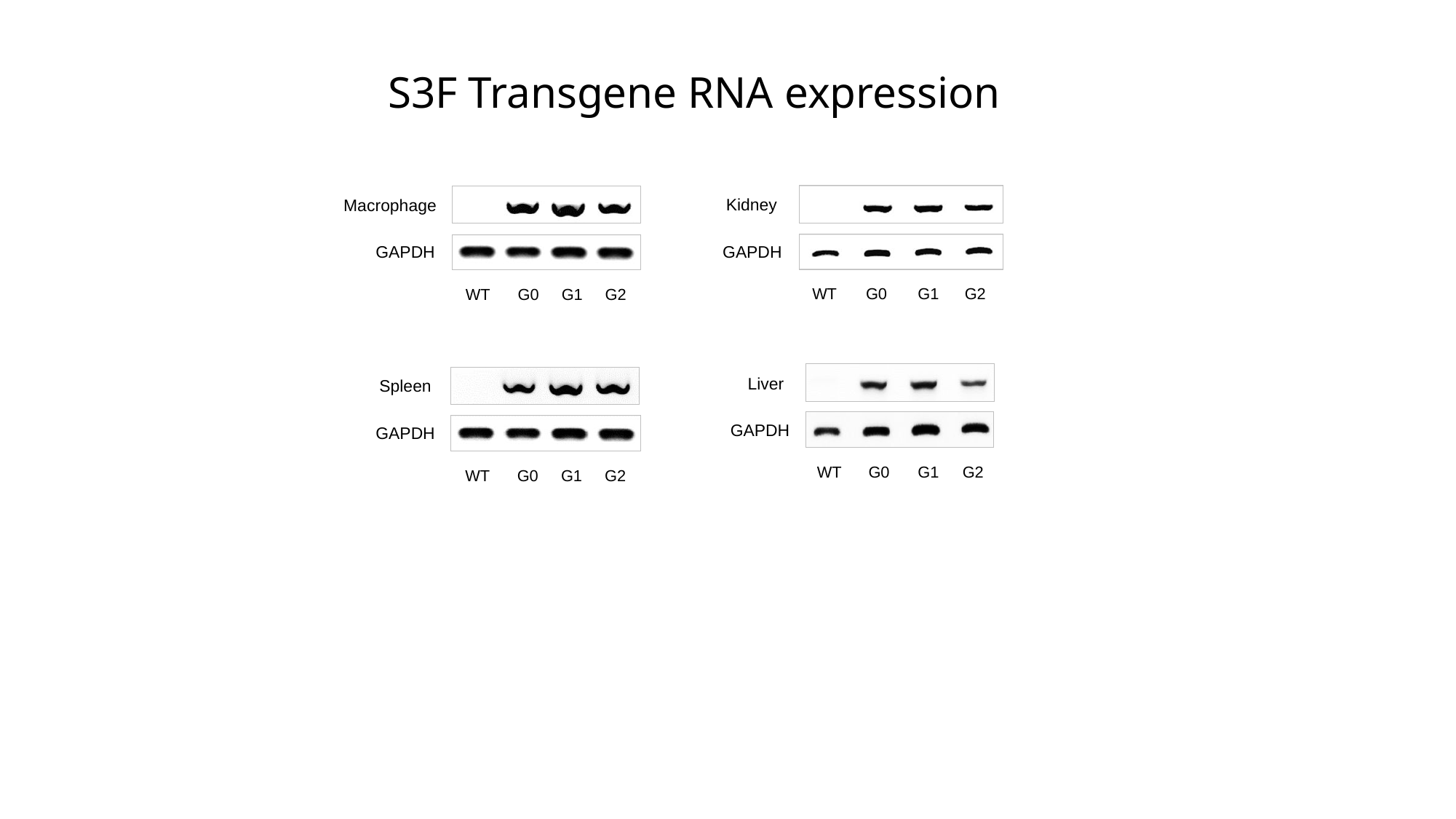

# S3F Transgene RNA expression
Kidney
Macrophage
GAPDH
GAPDH
WT
G0
G1
G2
WT
G0
G1
G2
Liver
Spleen
GAPDH
GAPDH
WT
G0
G1
G2
WT
G0
G1
G2
