# Supplementary material for: APOL1 renal risk variants promote cholesterol accumulation in tissues and cultured macrophages from APOL1 transgenic mice
Source: PLoS One. 2019 Apr 18;14(4):e0211559. doi: 10.1371/journal.pone.0211559 (PMC6472726; doi:10.1371/journal.pone.0211559)
Supplement: S7 Fig — Litter mate control (LC) or APOL1 mice with genotypes as shown were left intact (control) or were challenged to induced kidney injury with SAND (saline for drinking), Angiotensin II subcutaneous pump, nephrectomy, and 11-deoxycorticosterone subcutaneous pellets). Data are summarized as median and IQR. (PPTX) [file pone.0211559.s007.pptx]

## Slide 1
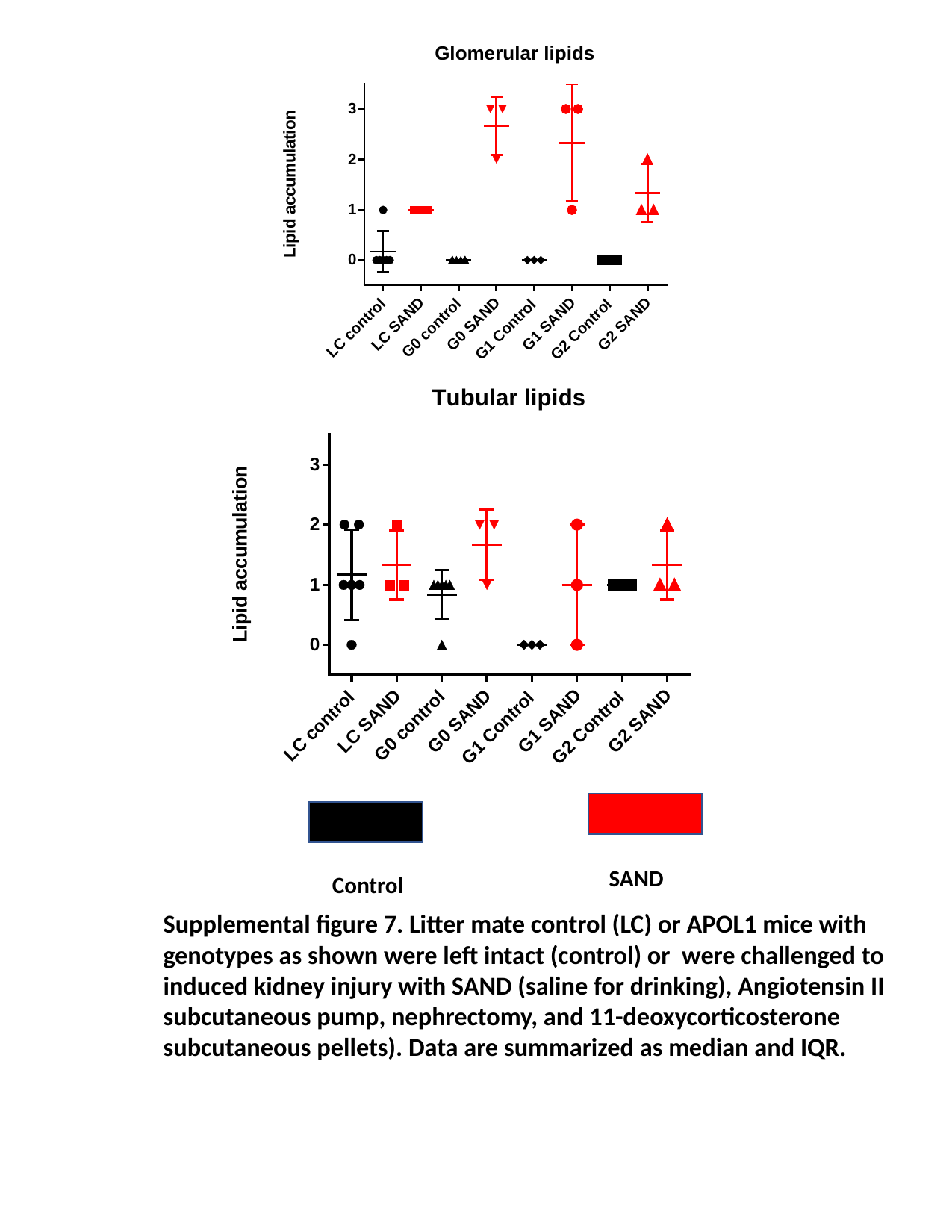

SAND
Control
Supplemental figure 7. Litter mate control (LC) or APOL1 mice with genotypes as shown were left intact (control) or were challenged to induced kidney injury with SAND (saline for drinking), Angiotensin II subcutaneous pump, nephrectomy, and 11-deoxycorticosterone subcutaneous pellets). Data are summarized as median and IQR.
