# Supplementary material for: APOL1 renal risk variants promote cholesterol accumulation in tissues and cultured macrophages from APOL1 transgenic mice
Source: PLoS One. 2019 Apr 18;14(4):e0211559. doi: 10.1371/journal.pone.0211559 (PMC6472726; doi:10.1371/journal.pone.0211559)
Supplement: S1 Table — (DOCX) [file pone.0211559.s009.docx]

**Supplemental Table 1. Primers for evaluation of macrophage polarization markers.**

| Gene | Sense | Antisense |
| --- | --- | --- |
| *Tnf* | CCCCAAAGGGATGAGAAGTT | CACTTGGTGGTTTGCTACGA |
| *Nos2* | GTTCTCAGCCCAACAATACAAGA | GTGGACGGGTCGATGTCAC |
| *IL12a* | CTGTGCCTTGGTAGCATCTATG | GCAGAGTCTCGCCATTATGATTC |
| *Arg1* | ACCATAAGCCAGGGACTGAC | AGGAGAAGGCGTTTGCTTAG |
| *Retnla* | CCAATCCAGCTAACTATCCCTCC | ACCCAGTAGCAGTCATCCCA |
| *Chi313* | AGAAGGGAGTTTCAAACCTGGT | GTCTTGCTCATGTGTGTAAGTGA |
