# Supplementary material for: APOL1 renal risk variants promote cholesterol accumulation in tissues and cultured macrophages from APOL1 transgenic mice
Source: PLoS One. 2019 Apr 18;14(4):e0211559. doi: 10.1371/journal.pone.0211559 (PMC6472726; doi:10.1371/journal.pone.0211559)
Supplement: Supplementary file 6 [file pone.0211559.s010.docx]

Genes correlated with APOL1 in highrisk group (2 APOL1 risk alleles)				
**EntrezGene**	**Name**	**Symbol**	**P-value**	**Association**
1071	Cholesteryl Ester Transfer Protein, Plasma	CETP	0.01	direct
19	ATP-binding cassette, sub-family A (ABC1), member 1	ABCA1	0.01	
64241	ATP-Binding Cassette, Sub-Family G (WHITE), member 8	ABCG8	0.01	
2194	Fatty acid synthase	FASN	0.02	
10577	Niemann-Pick Disease, Type C2	NPC2	0.04	
348	Apolipoprotein E	APOE	0.04	inverse
**Genes correlated with APOL1 in lowrisk group (0 APOL1 risk alleles)**				
**EntrezGene**	**Name**	**Symbol**	**P-value**	
7376	Nuclear Receptor Subfamily 1, group H, member 2	NR1H2	0.02	direct
123	Perilipin 2	PLIN2	0	inverse
2168	Fatty Acid Binding Protein 1, liver	FABP1	0	
9388	Lipase, endothelial	LIPG	0.01	
348	Apolipoprotein E	APOE	0.01	
6646	Sterol O-acyltransferase 1	SOAT1	0.02	
8309	Acyl-CoA Oxidase 2, Branched Chain	ACOX2	0.02	
345	Apolipoprotein C-III	APOC3	0.03	
10924	Sphingomyelin Phosphodiesterase, acid-like 3A	SMPDL3A	0.03	
440503	Perilipin 5	PLIN5	0.04	

(blue)	**cholesterol efflux related genes (specific)**
(pink)	**lipid dysmetabolism related genes (general)**
